# Effect of Dialyzable Leukocyte Extract on chronic cervicitis in patients with HPV infection

**Published:** 2017

**Authors:** MP Acosta-Rios, E Sauer-Ramírez, LJ Castro-Muñoz, M García-Solís, C Gómez-García, R Ocadiz-Delgado, A Martinez-Martinez, V Sánchez-Monroy, C Pérez-De la Mora, B Correa-Meza, DG Perez-Ishiwara

**Affiliations:** * PLaboratory of Molecular Biomedicine I, ENMyH, Instituto Politécnico Nacional, Mexico; ** Mexican College of Obstetrics and Gynecology Specialists, A.C. Mexico; *** Hospital General de Milpa Alta, Mexico City SSA, Mexico; **** Department of Genetics and Molecular Biology, Centro de Investigación y de Estudios Avanzados IPN, Mexico; ***** Department of Chemical and Biological Sciences, Universidad Autónoma de Ciudad Juarez, Mexico; ****** FARMAINMUNE, Azcapotzalco Mexico; ******* BIOXPORT, Azcapotzalco Mexico

**Keywords:** HPV, DLE, chronic cervicitis

## Abstract

The objective of the study was to assess the clinical, histopathological and immunochemical changes induced by dialyzable leukocyte extract (DLE) treatment in patients with chronic cervicitis associated to HPV infection. Fifty-four female Mexican patients diagnosed with chronic cervicitis, cervical intra-epithelial neoplasia grade 1 (CIN 1) and HPV infection were divided into two groups: patients treated with placebo and patients treated with DLE. Clinical and colposcopy evaluations were performed before and after treatments. Cervix biopsies were obtained to analyze histopathological features and to determine the local immunological changes by immunohistochemistry analyses. Placebo-treated patients showed no significant changes in the evaluated parameters. Interestingly, in DLE-treated patients, clinical manifestations of cervicitis diminished and 89% of them remitted the colposcopic lesions. Histological analyses of biopsies from DLE-treated patients showed a decreasing leukocyte infiltrate. Immunochemical analyses showed an increased expression of TGF-β, while expression of IFN-γ, PCNA, and IL-32 decreased. Our results suggest that DLE can stimulate innate immunity of cervical mucosae, diminishing chronic cervicitis in HPV-infected patients.

Trial registration: Register ISRCTN16429164

**Abbreviations:** HPV = Human Papilloma Virus; DLE = Dialyzable leukocyte extract

## Introduction

Chronic cervicitis in patients infected with persistent HPV genotypes has been associated with malignancy evolution and has been considered a very important factor triggering carcinogenesis; persistent inflammation increased cellular epithelium turnover, enhancing genetic alterations conjointly with the viral infection [**1**]. Patients with HPV infection usually present common symptoms of chronic cervicitis such as vaginal discharge and vaginal bleeding, dolor, vulvar or vaginal irritation and dysuria. Colposcopy findings include vaginal discharge, vaginal bleeding, cervical erythema, friability, erosion, and edema [**2**]. Histological analysis of the affected region, revealed besides cervicitis, several manifestations of HPV infection such as acanthosis, squamous metaplasia and koilocytic atypia [**3**]. Dialyzable leukocyte extract (DLE) has been reported as a modulator of the immune response, which up-regulated synthesis of molecules such as IL-2 and the activation and chemotaxis of macrophages and natural killer cells [**4**][**5**][**6**]. The DLE has been clinically tested in some diseases caused by viruses, fungi and parasites and has been used as adjuvant treatment for asthma, rheumatoid arthritis, atopic dermatitis, and respiratory infections [**7**][**8**][**9**][**10**][**11**][**12**][**13**][**14**][**15**]. Multiple evidence also suggest that the DLE administration increase CD4, CD8, CD16 and CD56 T-lymphocyte subpopulations in some pathologies [**16**][**17**]. In 2015, Rodriguez et al. specifically documented that DLE treatment in patients with low-grade cervical lesions, diminished or abolished HPV viral load, correlating it with clinical improvement [**18**]. Recently, we demonstrated that DLE promotes the expression of anti-inflammatory cytokines, avoiding NF-κB translocation to the nucleus in a rat osteoarthritis model and also that DLE modulates the inflammatory response in experimental autoimmune prostatitis [**19**][**20**].

Here, we evaluate the clinical effect of DLE treatment in CIN 1 patients with chronic cervicitis to down-regulate the inflammation process, registering the cervix tissue changes by colposcopy, histopathology, and immunochemical studies.

## Material and Methods

A total of 54 female Mexican patients with a cytological diagnosis of CIN 1, chronic cervicitis and HPV infection, were included. The protocol with the registration number 0152013 was reviewed and approved by the Ethics Committee of the National School of Medicine and Homeopathy from National Polytechnic Institute (Mexico). All the women included in the study signed the informed consent to be included in the protocol; chronically ill women who had diabetes, allergies, autoimmunity diseases, AIDS or other sexually transmitted diseases were excluded, likewise pregnant and menopausal women.

Clinical signs and symptoms of the women included in the study were evaluated. For colposcopy, the localization and description of the lesion were performed according to the number of cervical quadrants of the lesion covered; likewise, the area of the lesion was described according to the cervical area percentage [3]. Then, biopsies were processed as described below for histopathological analysis, selecting for the study those with confirmed chronic cervicitis and CIN 1 diagnosis. HPV infection was corroborated by PCR using the DNA extracted from 10µm thick biopsy sections using the QIAamp DNA Mini Kit (QIAGEN, USA) and the multiplex PCR Kit for Human Papilloma Virus (Maxim Biotech, Inc, USA) according to manufacturer's instructions.

Selected patients were randomly divided into two groups: placebo and DLE-treatment groups. DLE was prepared by repeated freezing and thawing of leukocytes isolated from Crocodylus moreletti. The dialyzed extract of leukocytes was adjusted by protein concentration and stored lyophilized until use. One unit of DLE containing 0.100 mg of extract and Glycine (150 mg) was reconstituted in 2 ml of bi-distilled sterile water. Otherwise, one unit of placebo only containing Glycine (150 mg) was also reconstituted in 2 ml of bi-distilled sterile water. Patients from both placebo and DLE-treated groups were orally administered the solution at every 72 hours for four weeks.

At the end of the treatment patients were clinically evaluated again and assessed by colposcopy, and cervical samples were taken for both histopathological and immunohistochemical analyses.

Hematoxylin and eosin (H&E) staining (Sigma Aldrich, St Louis, MO, USA) was performed to analyze the histopathological characteristics of the lesions. Briefly, tissue sections (3 μm thick) were cut using the microtome (American Optical rotary microtome 820). Then, histologic sections were immersed in xylene to remove excess paraffin. Tissue sections were rehydrated by passing through a decreasing concentration gradient of alcohol and water baths (100%, 90%, 80%, and 70%). Subsequently, the tissue sections were immersed in hematoxylin for 10 minutes and rinsed in tap water until the sections exhibited a blue coloration. The tissue sections were immersed in 1% alcoholic acid (1% HCl in 70% alcohol) for 5 minutes. Then, the histological sections were washed in running water, placed into the eosin for 30 seconds and treated with another series of alcohol baths, in increasing order (70%, 95%, and 100%). Finally, the sections were left in xylol for 10 minutes and mounted on electrocharged glass slides (Fischer Scientific, USA). Protein detection was performed using the Mouse/Rabbit PolyDetector HRP/DAB Detection System (Bio SB, USA) according to the manufacturer's instructions. Briefly, the tissues were rinsed with 10% formaldehyde in phosphate-buffered saline (PBS), and epitope retrieval was performed in a pressure cooker (121°C, 20 lb. of pressure) using the ImmunoRetriever Citrate Solution (Bio SB, USA) for 15 min. The slides were cooled at room temperature (30 min), and the tissues were treated with PolyDetector Peroxidase Block quenching buffer (Bio SB, USA) for 1 minute. After three PBS washes, the sections were incubated at 4°C for 16 hr with monoclonal antibodies against PCNA, TGF-β and IFN γ proteins (Santa Cruz Biotechnology, USA) and a polyclonal primary antibody against IL-32, (Abcam, USA). Antibodies were used at a 1:50 dilution. After four PBS washes, the slides were incubated with the secondary antibody (PolyDetector HRP label; Bio SB, USA) for 30 min. After three PBS washes, the sections were incubated with the appropriate substrate (PolyDetector DAB chromogen; Bio SB, USA), counterstained with hematoxylin and mounted in GVA-mount reagent (Zymed, USA). Negative controls were performed without the primary antibody incubation.

For digital immunohistochemical analyses, all photomicrographs were obtained using a DFC290 HD digital camera (Leica Microsystems, USA), processed in the PhotoImpact software (Ulead PhotoImpact SE ver. 3.02; Ulead Systems, U.S.A.), and digitally analyzed using the Image-ProPlus Analysis Software (Version 4.5.0.19, Media Cybernetics, Inc., and U.S.A). The chromogen quantity was determined by calculating the norm of the matrix file for each image using the “Measure” tool. This allows pixels of similar “color” immediately adjacent to the index pixel to be included for analysis. All pixels falling within the selected threshold parameters were quantified, recorded, and used to generate the graphs. The file for the control image is generated similarly: The biopsy control slide is acquired and treated identically as the experimental slide except that negative controls were included. Likewise, digital analyses were performed counting 16 representative epithelial cervical areas from each sample biopsy. The % labeling index was obtained by the formula: labelling index = (100%) *(positive signal) / total signal.

## Results

Fifty-four Mexican women with chronic cervicitis, CIN 1 and HPV infection with the ages ranging from 20 to 65 years old were included in the study. From the total, 19 did not present clinical symptoms, and 35 had gynecological symptoms such as white vaginal fluid, pelvic pain and recurrent urinary tract infections. The post-treatment symptoms of the patients receiving placebo essentially reported no changes, while 90% of the patients treated with DLE had resolved most of the symptoms (**Table 1**).

**Table 1:** Clinical evaluation of experimental groups

Symptoms	Number of Patients			
	DLE treatment		Placebo treatment	
	Before	After	Before	After
White vaginal fluid	11	1	8	8
Bleeding	0	0	0	0
Pelvic Pain	4	1	2	2
Recurrent urinary tract infection	7	0	3	3

**Fig. 1:**
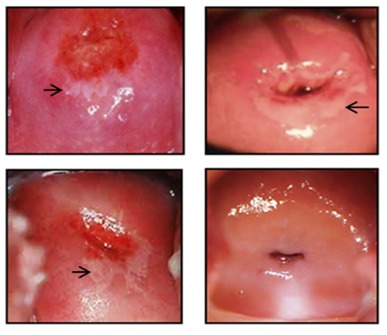
Comparative colposcopy images of the cervix from representative patients before and after placebo or DLE treatments. The images are representative of patients from each group. The arrows indicate the resolution of acetowhite lesion after the treatment with DLE

Colposcopic analyses before and after treatments were performed documenting the anatomical localization and size of the lesions. Before treatment, a thin acetowhite epithelium with irregular borders, fine punctuation, and a fine mosaic pattern was observed (**Fig. 1**). After the therapeutic intervention, the lesions observed in patients from the placebo group did not display differences. In contrast, in the DLE-treated patients, we observed normal colposcopy images in 89% of cases, showing a cervix with a uniform pink color without visible injuries (**Fig. 1**). After one year of colposcopy monitoring, patients did not show recurrence or persistence of the lesions.

**Fig. 2:**
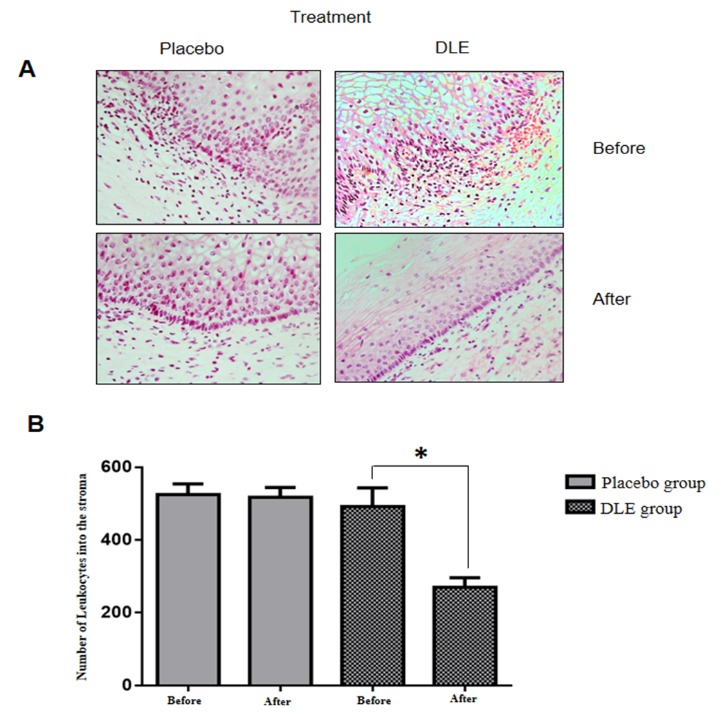
Representative images of histopathological features of cervix tissues. (A) H-E staining of cervix samples from patients before and after treatment with placebo or DLE. The decrease of the inflammatory process in patients treated with DLE was observed. (B) Semiquantitative analyses of infiltrating leukocytes into the stroma. The asterisk marks statistically significant differences (p<0.05). The analyses were performed counting 16 representative epithelial cervical areas from each sample biopsy.

Before therapeutic intervention with DLE, H&E staining analysis of the cervical tissues from both groups showed histological alterations including hyperchromatic and pleomorphic cells with abnormal chromatin distribution; vascular congestion; and important inflammatory process with edema and leukocyte infiltration in the stroma (**Fig. 2**A). The dysplastic cells occupied the lower third of the epithelium, classifying it as a low-grade lesion (CIN 1). No changes were observed in the biopsies obtained from the placebo group after treatments. However, the DLE treated group showed important differences, the histological architecture of the cervical epithelium was more conserved, with discrete reduction of dysplastic cells; the basal membrane was continuous and well defined; there was a reduction of stromal edema and vascular congestion by approximately 40%; the parabasal, middle and superficial areas were observed with a better cell differentiation (**Fig. 2**A). Likewise, in the stroma, we observed a statistically significant reduction of leukocyte infiltration of about 50% on average (**Fig. 2**B).

## Treatment

**Fig. 3:**
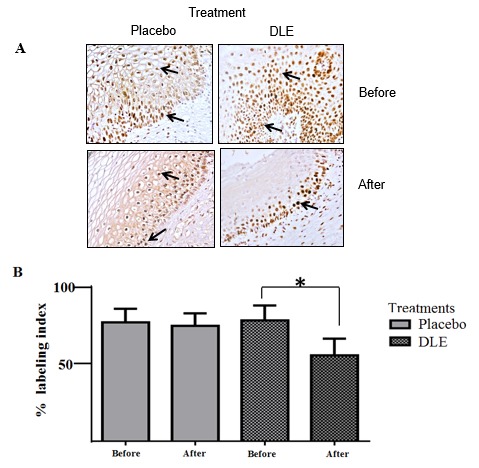
Immunohistochemical detection of PCNA. (A) Representative images of cervix biopsies from a patient before and after treatments. The arrows indicate the nuclear PCNA immunolabeling. The decrease of immunolabeling of PCNA in patients treated with DLE was observed (B) Semiquantitative analysis of PCNA detection. The asterisk marks statistically significant differences (p<0.05). The analyses were performed counting 16 representative epithelial cervical areas from each sample biopsy

Considering the histopathological changes induced by DLE, we measured the cell proliferation levels in cervical epithelia. Immunohistochemical analyses for Nuclear Proliferating Cell Antigen (PCNA) protein showed positive staining in the basal and parabasal layers of cervical tissues of patients from both groups before treatment and tissues from the placebo-treated group (**Fig. 3**A). In contrast, samples obtained from DLE-treated patients showed a decreased PCNA staining, detecting it mainly in the basal layer. The immunostaining decrease observed was approximately 19% on average, and it was statistically significant, (p<0.05) (**Fig. 3**B).

**Fig. 4:**
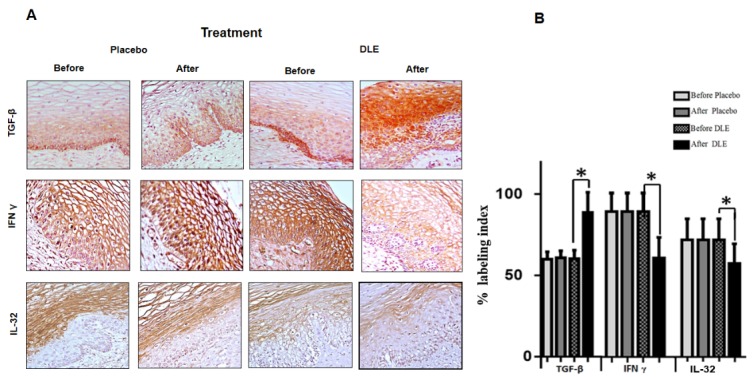
(A) Immunohistochemical detection of TGF-β, IFN γ and IL-32. Representative images of cervix biopsies from a patient before and after treatments. The increase of immunolabeling of TGF-β and the decrease of INF-γ and IL-32 in patients treated with DLE were observed (B) Semiquantitative analyzes of immunohistochemical staining for TGF-β, IFN γ and IL-32. The asterisk marks statistically significant differences (p<0.05). The analyses were performed counting 16 representative epithelial cervical areas from each sample biopsy

We searched for the detection of TGF-β, IFNγ and IL-32 cytokines implicated in both the HPV infection and cervicitis process. Results showed that cervical tissues from the placebo group did not display significant differences in the detection patterns of TGFβ, IFNγ and IL-32, before and after treatment. Conversely, cervixes from DLE-treated patients showed some differences. Before treatment, the immunostaining for TGF-β was limited to the middle and parabasal layers of the cervical epithelium, while after DLE treatment the immunostaining was evident in almost all epithelium, increasing with 38% on average (p<0.05) (**Fig. 4**A,B).

IFN-γ detection was observed before DLE treatment throughout the epithelium, being more intense in the middle epithelial layer. Interestingly, after treatment, we found that staining significantly decreased in all epithelial layers, decreasing the labeling index with 35% on average (**Fig. 4**A,B).

Finally, IL-32 was detected before treatment in the medial and superficial layers of cervical epithelia, while after DLE treatment we observed a significant decrease, detecting a faint signal mainly in the middle layer of cervical epithelium (**Fig. 4**A). Statistical analysis showed that the decrease was significant, diminishing with approximately 30% on average (p<0.05) (**Fig. 4**B).

## Discussions

Chronic cervicitis in patients infected with persistent HPV genotypes is a very important factor triggering viral-induced carcinogenesis [**21**]. An effective mucosae immune response has been related to an effective priming of the adaptive immune response facilitating viral resolution. Thus different immune modulators have been studied to enhance or to promote the innate immune response [**22**][**23**]. Among them, the dialyzable leukocytes extract (DLE) has been used in viral infections, and it could be used as a putative co-adjuvant strategy to stimulate the mucosal immunity [**4**]-[**20**]. In the present work, we evaluated the effect of DLE treatment to diminish chronic cervicitis in CIN 1 HPV patients, focusing our evaluation on the modification of some important cervical mucosae immunity factors for anti-inflammatory and anti-viral responses. 

The clinical symptoms of 54 patients with a diagnosis of chronic cervicitis and HPV infection lesions showed that 64% of them displayed symptoms associated with HPV infection and cervicitis; these data are in concordance with the meta-analysis described by Gillet in 2011 [**24**], reporting a positive association between bacterial vaginosis and uterine cervical HPV infection.

Interestingly, we found that after administration of one unit of DLE at every 72 hours for four weeks, in most of the patients, cervicitis symptoms diminished; colposcopically, lesions associated with cervicitis and HPV infection were solved in 89% of the cases. These results are in concordance with the study that Rodriguez et al. conducted in 2015, in which they found that patients with low-grade intraepithelial squamous cervical lesions treated with human DLE decreased the size or produced the absence of lesions in 79% of the patients [**18**]. However, we found important differences in DLE doses employed and the administration scheme of both studies. Our results showed colposcopic resolution of cervical lesion in 89% of the patients after 4 weeks of oral treatment with Crocodylus moreletii DLE of 0.3 mg per week, while Rodriguez et al. used a combined therapy of human DLE of 2.2 mg per week, orally for 5 weeks and 2.2 mg at every 72 hr for two weeks used topically. In fact, authors repeated this schedule of treatment when a persistent lesion was found during colposcopy examination. These results suggest that Crocodylus moreletii DLE was more effective than human DLE to treat CIN 1 lesions. Findings from both groups contrast with results obtained by Luciani et al. in 2008 using cervical cryotherapy, an invasive method, in which even though it was effective to diminish the clinical symptoms in 88% of the patients with CIN diagnosis, it could have important side effects such as a profuse watery vaginal discharge for at least 6 weeks [**25**].

Before treatment, the histopathological analysis of tissue of most samples showed an intense leukocyte infiltration at the cervical stroma, similarly with the findings described by Castle et al. in 2001 [**26**] and Mirzaie et al. in 2014 [**27**]. However, after the DLE treatment, a 50% reduction of leukocyte infiltrate was found. The anti-inflammatory effect of DLE was previously reported in 2004 by Orozco and et al., who studied patients diagnosed with atopic dermatitis, reporting that after 10 weeks of DLE treatment, the number of eosinophils was reduced, modifying the inflammatory mediators [**9**]. In 2005, Ojeda et al. reported that the DLE modulate the production of proinflammatory cytokines in leukocytes activated by the bacterial cell wall components, lipopolysaccharide, lipoteichoic acid, and peptidoglycan, suppressing the production of TNFα, as well as the NF-κB activity inhibition [**4**]. In our work, we also found that after DLE treatment, a decrease in stromal infiltrating leukocytes could be observed, correlating it with clinical remission of symptoms, strongly suggesting that the mucosal immunity response is enhanced by the DLE treatment, diminishing the inflammatory process. In 2015, Rodriguez et al. reported that the treatment with human DLE in patients with CIN1 decreased viral load [**18**].

This result is related to our immunohistochemical analysis of the PCNA protein, where we observed a decrease in the cellular proliferation associated with the viral presence.

Thus, we focused our analysis on the evaluation of some specific effector molecules related to the inflammatory/anti-inflammatory processes and the viral persistence. In the cervical mucosae, the microenvironment induced by HPV infection promoted a down-regulation of antigen presentation and the inhibition of activation and migration of Langerhans cells [**28**], also promoting the imbalance of anti- and pro-inflammatory cytokines, generating inflammation [**29**][**30**]. It has been reported that the low expression of TGF-β diminishes macrophages and monocytes chemo-attraction, allowing progression to invasive cervical cancer [**31**]. Our results suggest that DLE treatment could modify the inflammatory cervix microenvironment, promoting the expression of TGF-β, and probably triggering the synthesis of anti-inflammatory cytokines involved in local immune mechanisms of the cervix. In 2014, Garcia et al. described that DLE directly activated monocytes through TLR-2, suggesting that part of the immunomodulatory properties of DLE could be attributed to TLR-2 activation on monocytes for the control of infectious diseases [**5**].

On the other hand, it has been documented that IFN-γ expression increases in relation to the grade of cervical injury [**32**]. These reports are in concordance with our findings showing that before the intervention, an important detection of INF-γ was observed, while after DLE treatment, the INF-γ expression diminished.

Finally, we looked for the detection of IL-32, so it has been documented that it is overexpressed in HPV-positive cervical cancer cells [**33**], although no evidence has been reported in earlier pre-neoplastic lesions. Besides, in 2014, Zeng et al. demonstrated that IL-32 overexpression contributes to invasion and metastasis in lung adenocarcinoma, promoting cell migration via transactivation of nuclear transcription factor NF-κB pathway [**34**]. Interestingly, in this work, the expression of IL-32 in the cervix of patients treated with DLE decreased, suggesting that this effect could also diminish the risk of disease progression. In this context, our group reported in 2016 that the administration of DLE promotes the synthesis of anti-inflammatory cytokines in an osteoarthritis disease model, inhibiting the translocation of nuclear transcription factor NF-κB to the nucleus [**18**]. Taking together the results presented here, we can suggest that DLE treatment could modify the inflammatory status of the cervix mucosae via down-regulation of IL-32.

## Conclusion

In conclusion, our results suggest that DLE treatment could modulate the innate immune response in cervical mucosae, diminishing the inflammatory effectors and the clinical symptoms in CIN 1 HPV patients with chronic cervicitis. 

## Acknowledgements

We would like to thank Lic. Polete Ramirez (Hospital Tláhuac, México) for technical support. This work was supported by Consejo Nacional de Ciencia y Tecnología (México). During this work, MPAR was the recipient of a fellowship from CONACyT (Grant number: 324925).

## Conflict of interest

The authors declare no conflict of interest.

## Supplementary information

Fig. S1. Representative IFTR profiles of human and crocodile DLE.

Table S1. Patients' demographics.
