# Supplementary figures and images for: Effect of Dialyzable Leukocyte Extract on chronic cervicitis in patients with HPV infection
Source: J Med Life. 2017 Oct-Dec;10(4):237–43. (PMC5771637)

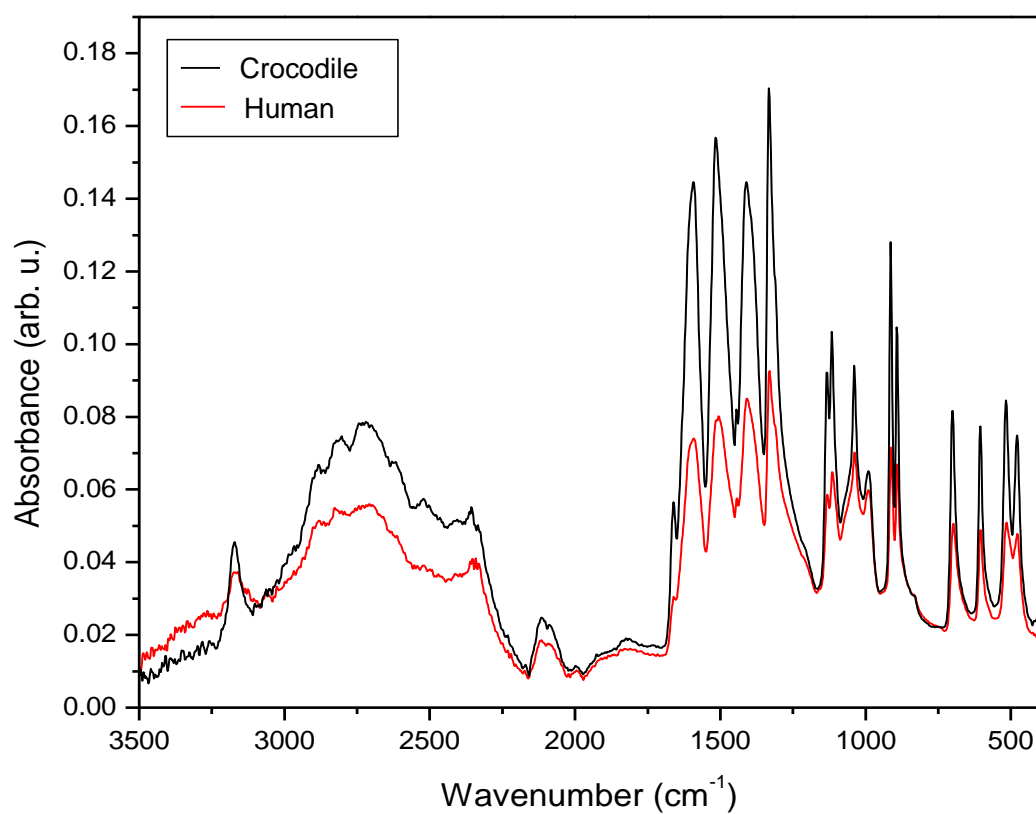

**Figure S1** Representative IFTR profiles of human and crocodile DLE.

Supplement: Fig. S1. Representative IFTR profiles of human and crocodile DLE. [file JMedLife-10-237-s001.pdf]
