# Supplementary material for: Effect of Dialyzable Leukocyte Extract on chronic cervicitis in patients with HPV infection
Source: J Med Life. 2017 Oct-Dec;10(4):237–43. (PMC5771637)
Supplement: Table S1. Patients' demographics. [file JMedLife-10-237-s002.pdf]

Table S1. Patients' demographics.

NUMBER OF THE PATIENT'S	PATIENT'S KEY	PATIENT'S AGE	COLPOSCOPIC AND HISTOPATHOLOGICAL DIAGNOSTIC	TREATMENT	TIME SINCE DIAGNOSIS	VIRAL GENOTYPING BY PCR
1	E-133-12	29	CIN-1	DLE	2 MONTH	18,31,
2	E-122-1	24	CIN-1	DLE	2 MONTH	31,16,18
3	E-149-12	31	CIN-1	DLE	3 MONTH	18,11
4	E-176-12	38	CIN-1	DLE	2 MONTH	16,18
5	E-119-12	43	CIN-1	DLE	3MONTH	16,31
6	S-246	25	CIN-1	DLE	2 MONTH	16,18
7	E-112-11	33	CIN-1	DLE	2MONTH	16,18,
8	E-156-11	41	CIN-1	DLE	2MONTH	31, 18
9	E-149-11	39	CIN-1	DLE	3 MONTH	18, 31
10	E-88-11	32	CIN-1	DLE	2MONTH	16, 18
11	E-124-11	30	CIN-1	DLE	2 MONTH	16 ,18 ,
12	E-167-11	28	CIN-1	DLE	2 MONTH	16, 18
13	E-186-11	31	CIN-1	DLE	2 MONTH	6,31,16
14	E-296-12	37	CIN-1	DLE	3 MONTH	52,31
15	E-311-12	38	CIN-1	DLE	2 MONTH	52, 16,
16	E-309-12	45	CIN-1	DLE	3MONTH	52,31
17	E-300-12	27	CIN-1	DLE	2 MONTH	6,11,31
18	E-313-12	36	CIN-1	DLE	2MONTH	11,31
19	E-325-12	38	CIN-1	DLE	2MONTH	52,31
20	E-302-11	32	CIN-1	DLE	3 MONTH	16,18
21	E-292-12	29	CIN-1	DLE	2MONTH	11, 16
22	E-312-12	39	CIN-1	DLE	2 MONTH	31,16
23	E-575-12	41	CIN-1	DLE	2 MONTH	52,33
24	E-577-12	39	CIN-1	DLE	3 MONTH	,58,31,11
25	E-609-12	47	CIN-1	DLE	2 MONTH	16, 18
26	E- 714-12	33	CIN-1	DLE	2 MONTH	,16,18
27	E-757-12	38	CIN-1	DLE	3 MONTH	52, 18,
28	E-780-12	27	CIN-1	DLE	2 MONTH	58, 31,
29	E-227-12	44	CIN-1	DLE	3MONTH	33, 11,
30	E-106-12	36	CIN-1	DLE	2 MONTH	,16,18
31	E- 84-12	45	CIN-1	DLE	2MONTH	16,33
32	E-92-12	37	CIN-1	DLE	2MONTH	16,18
33	E-135-12	46	CIN-1	DLE	3 MONTH	33,16
34	E- 83-12	39	CIN-1	DLE	2MONTH	16,18,
35	124	32	CIN-1	PLACEBO	2 MONTH	31,16
36	53	27	CIN-1	PLACEBO	3 MONTH	11,31,
37	430	31	CIN-1	PLACEBO	2MONTH	52,31
38	178	26	CIN-1	PLACEBO	3 MONTH	58,33
39	136	38	CIN-1	PLACEBO	2 MONTH	11, 18
40	134	49	CIN-1	PLACEBO	2 MONTH	11,18
41	118	29	CIN-1	PLACEBO	3 MONTH	52, 18,
42	137	42	CIN-1	PLACEBO	2 MONTH	11, 31,
43	120	36	CIN-1	PLACEBO	3MONTH	33, 16
44	146	31	CIN-1	PLACEBO	2 MONTH	16,18,6
45	152	43	CIN-1	PLACEBO	2MONTH	16, 6
46	138	37	CIN-1	PLACEBO	2MONTH	16,18
47	93	39	CIN-1	PLACEBO	3 MONTH	31,16
48	154	41	CIN-1	PLACEBO	2MONTH	16,18,
49	66	28	CIN-1	PLACEBO	2 MONTH	16,31,
50	8	37	CIN-1	PLACEBO	3 MONTH	52,31
51	106	29	CIN-1	PLACEBO	3 MONTH	58,31
52	809	38	CIN-1	PLACEBO	2MONTH	16, 18
53	715	27	CIN-1	PLACEBO	3 MONTH	33,18
54	775	36	CIN-1	PLACEBO	2 MONTH	52, 18,
